# Two-weeks repeated-dose oral toxicity study of *Pediococcus acidilactici* J9 in a mice model

**DOI:** 10.1186/s12866-020-02055-4

**Published:** 2020-12-09

**Authors:** Mijung Lee, Jin-Young Chung, Ka Yeun Kim, Wooseok Im, Manho Kim

**Affiliations:** 1 Department of Neurology, Biomedical Research Institute, Seoul National University Hospital, 101 Daehak-ro, Jongno-gu, Seoul, 03080 South Korea; 2 Department of Veterinary Internal Medicine and Geriatrics, College of Veterinary Medicine, Kangwon National University, Gangwon-do, South Korea; 3 Department of Psychology, Fordham University, New York, NY USA; 4 Neuroscience Research Institute, Seoul National University College of Medicine, Seoul, South Korea; 5 Protein Metabolism Medical Research Center, College of Medicine, Seoul National University Hospital, Seoul, South Korea

**Keywords:** Pediococcus acidilactici J9, Repeated two-week oral dose toxicity, Mouse, Helicobacter pylori, AGS cell

## Abstract

**Background:**

Helicobacter pylori (*H. pylori*) is an important pathogen that causes chronic gastritis and peptic ulcer, and is related to the development of gastric carcinoma. Several chemicals, including antibiotics, have been used to eradicate *H.pylori*. However, more studies are yet requred to accomplish a sufficient therapy. *Pediococcus acidilactici* (*P. acidilactici*) J9 were studied for inhibition of binding of *H.pylori* binding to human gastric cell lines. This study was performed in order to investigate the repeated-dose toxicity of *P. acidilactici* J9 in male and female mice.

**Results:**

C57BL/6 male and female *Mus musculus* were divided into four groups (*n* = 10 in each group). *P. acidilactici* J9 was administered daily by oral injection of vehicle control at dosage levels to a low-dose group (500 mg/kg/day), middle-dose group (1000 mg/kg/day), and high-dose group (2000 mg/kg/day) for 2 weeks. After 14 days of exposure, the blood biochemistry and hematology were investigated, along with a histopathology exam. There were no bacterial-related deaths or abnormal clinical signs in either gender of mouse. The data was observed during the period in terms of body weight, food intake, and water consumption. Also, no alterations in organ weights upon administration of *P. acidilactici* J9 alone were observed. The adhesion and growth of *H. pylori* were inhibited by a 24 h treatment of *H. pylori* and *P. acidilactici* J9 on adenocarcinoma gastric (AGS) cells, which are gastric cancer cells. Compared to the control group (AGS cell and *H. pylori*), the number of *H. pylori* analyzed by FACS significantly (*p* < 0.01) decreased after incubation of AGS cell with *P. acidilactici* J9 for 24 h.

**Conclusions:**

These results suggest that the oral application of *P. acidilactici* J9, up to a dosage level of 2000 mg/kg/day, causes no adverse effects in both male and female mice. *P. acidilactici* J9 inhibits the adhesion of *H.pylori* to AGS cancer cells. When used as probiotics, *P. acidilactici* J9 may help decrease the occurrence of gastritis and reduce the risk of *H.pylori* infection with promising safety issues.

**Supplementary Information:**

The online version contains supplementary material available at 10.1186/s12866-020-02055-4.

## Background

Lactobacillus is a gram-positive microorganism that utilizes carbohydrates as the energy source and produces organic acids like lactic acid and acetic acid as final products. It is used industrially in various fermented products, like fermented vegetables and dairy products, which are broadly involved in the everyday life [1, 2]. Lactobacillus determines the flavor of fermented foods and the characteristics of fermented products, and it plays a critical role in the food preservation. This occurs by extending its shelf life via production of active antibiotic materials like organic acid and bacteriocin. Moreover, various function in the human body is also reported, such as the suppression of intestinal noxious bacteria, the decrease of blood cholesterol levels, anticancer effect, reinforcement of immune function, etc. Because of the Lactobacillus, as a probiotics, intakes living strain, it attracts attention as an antibacterial preparation that solves the residual tolerance problems, in addition to being recently utilized as a healthy functional food [3, 4].

*Helicobacter pylori* is a macroaerophilic gram-negative bacteria that causes chronic gastritis, peptic ulcer, and presumable gastric cancer. Accumulated evidence demonstrates that the eradication of these bacteria resolves *H.pylori*-associated disease [5]. Multicenter studies have shown that triple therapy via a proton pump inhibitor (PPI), clarithromycin, and either amoxicillin or metronidazole (all taken twice daily). This therapy is among the most effective approaches to *H. pylori* eradication [6]. However, 5–10% of *H. pylori* strains are reportedly resistant to clarithromycin [7]. In addition, there was a study noted a clarithromycin-resistant mutation in 63% of *H. pylori* strains from patients in whom treatment with a regimen including clarithromycin was unsuccessful [8]. The treatment of *H. pylori* infection with antibiotics does not eradicate the organism and is also often accompanied by deleterious side effects [9]. Thus, although many experts believe that “untreatable” *H. pylori* is just ill-treated *H. pylori*, no clinical trial. To the best of our knowledge, *H. pylori* has not yielded a treatment that provides 100% eradication [10].

Recently, probiotic lactic acid bacteria (LAB) have been reported to control *H. pylori*. Also, several studies have examined the efficacy of various probiotic preparations for *H. pylori* eradication with and without co-interventions [11]. Moreover, a number of clinical trials have been undertaken to test the hypothesis that probiotic bacteria inhibits *H. pylori* infection [12]. Probiotics inhibit enteric pathogens such as *Salmonella*, *Shigella*, and *Citrobacter rodentium* in both in vitro and in vivo [13, 14], and potential clinical benefits in preventing or resolving gastrointestinal diseases have been demonstrated [15, 16]. These microorganisms provide gut protection through several mechanisms, including decreasing luminal pH by producing lactic acid [17, 18] and competing with gut pathogens for host surface receptors [19]. Nonetheless, it has been shown that probiotics may inhibit *H. pylori* growth, independent of pH and lactic acid levels [20].

We have focused on *P. acidilactici* J9, originated from Kimchi and Cheonguk-jang [21], which produces bacteriocin [22] that is known as a considerably widely used antibacterial agent. Recently, *P. acidilactici* J9 is attracting attention as probiotics because it has interesting properties, such as resistance to heat, cold, pH, and proteolytic treatments, which are the required properties for probiotics [23]. It is reported that *P. acidilactici* has the ability to survive through gastrointestinal tract passage, can survive from a drying process and during storage at 4°C for 60 days [24]. Moreover, *P. acidilactici* has been reported to have in vitro acvitity against *H. pylori* [25].

However, a systematic study on its repeated oral administration toxicity has not been reported yet. Therefore, we have investigated the toxicity of this new probiotics and its effect on the inhibition of growth of *H. pylori*. In vitro assays were carried out to determine whether the combination of *P. acidilactici* J9, and its adhesion to gastric cells thus impacting gastric acidity, inhibit the growth of *H. pylori* [26]. The current therapeutic regimen for *H. pylori* aims to eliminate bacterial growth with antibiotics and this reduces the total acidity of gastric acid.

In this study, repeated toxicity tests are performed as the stability test using mice of C57BL/6 type under the “standard of toxicity test for medicine and medical supplies (Korea food and drug administration notification No. 1999-61)”. We also demonstrated in in vitro models that *P. acidilactici* J9 in combination have beneficial effects similar to those of antibiotic therapy on *H. pylori*-infected gastric epithelium.

## Results

### Oral toxicity study of *P. acidilactici* J9 in a mice model: death rate and normal symptoms

*P. acidilactici* J9 was administrated by oral injection for 2 weeks and the Table 1 show the death rate and the normal symptoms of males and females observed for 2 weeks. During the experiment, experimental mice were observed at regular times, and no death was observed in the male and female administration group (Table 1). Also, during all of the experiment, in every administration group – low dose (500 mg/kg/day), medium dose (1000 mg/kg/day), and high dose (2000 mg/kg /day) - including the control group, no specific adverse symptoms are observed. In this study, a dose of 2000 mg/kg, which is a maximum dose of oral administration toxicity test, did not generate abnormal symptoms. It thus seems that the minimal lethal dose of this experimental materials exceeds 2000 mg/kg/day in both male and female.

**Table 1:** Mortality and clinical signs of male and female (mice) treated orally with *P. acidilactici* J9 for 14 days

Sex	Dose (mg)	No. of animal	Days after treatment				Final mortality	Clinical signs
			0	7	14	End		
Male	Control	5	0/5	0/5	0/5	T.S	0/5	NAD
	500	5	0/5	0/5	0/5	T.S	0/5	NAD
	1000	5	0/5	0/5	0/5	T.S	0/5	NAD
	2000	5	0/5	0/5	0/5	T.S	0/5	NAD
Female	Control	5	0/5	0/5	0/5	T.S	0/5	NAD
	500	5	0/5	0/5	0/5	T.S	0/5	NAD
	1000	5	0/5	0/5	0/5	T.S	0/5	NAD
	2000	5	0/5	0/5	0/5	T.S	0/5	NAD

Values are expressed as the numbers of dead animals/total numbers of animals

Values are expressed as number of animals with the sign/number of animals examined

*T*.S terminal sacrifice, *NAD* no abnormalities detected

### Oral toxicity study of *P. acidilactici* J9 in a mice model: changes in body weight

**Fig. 1:**
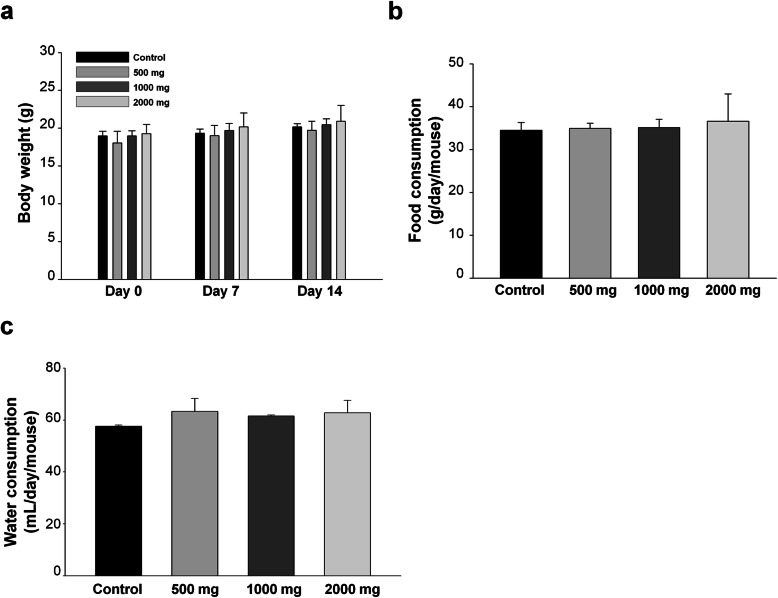
Changes in body weight and intake of C57BL/6 mice which treated *P. acidilactici* J9. Dosage is set by the standard of MFDS. Maximum dosage is set to 2000 mg/kg/day for both male and female, and with the geometric ratio of 1/2, low dose group, medium dose group, and high dose group are set by per body weight (kg) respectively. **a** For every animal, change of weight is measured just before the administration of test materials once a week at certain time during 2 weeks. **b, c** Intake of feeds and water is measured and calculated once a week. Feeds; solid feeds for laboratory animal are freely offered, and drinking water; filtration-purified water is also freely offered. *N* = 10 samples per group

*P. acidilactici* J9 was orally administrated for 2 weeks with varying concentrations and the changes in body weight are shown in Table 2. Changes in body weight during the whole period of experiment were negligible for the control group, low dose group (500 mg/kg/day), medium dose group (1000 mg/kg/day), and high dose group (2000 mg/kg/day). Additionally, from the date of administration of experimental materials to the end of the experiment, there was a normal weekly increase in body weight in the control group and the administration group (Fig. 1a).

**Table 2:** Body weight changes of male and female (mice) treated orally with *P. acidilactici* J9 for 14 days

Sex	Dose (mg)	Body weight (g)		
		Days after treatment		
		0	7	14
Male	Control	19.07 ± 0.80^NS^	19.64 ± 0.95 ^NS^	20.62 ± 1.13 ^NS^
	500	18.79 ± 1.32	19.40 ± 1.53	20.22 ± 1.39
	1000	19.21 ± 0.70	19.90 ± 1.04	20.59 ± 1.11
	2000	19.08 ± 0.92	19.89 ± 1.50	20.83 ± 1.79
Female	Control	16.60 ± 0.96^NS^	16.67 ± 0.77^NS^	17.79 ± 0.77^NS^
	500	16.64 ± 0.64	16.94 ± 0.40	17.49 ± 0.47
	1000	16.41 ± 0.64	16.78 ± 0.71	17.37 ± 0.79
	2000	16.30 ± 0.79	16.16 ± 0.96	16.92 ± 0.85

Values are expressed as mean ± SE (*n* = 5)

*NS* not significantly different among groups

### Oral toxicity study of *P. acidilactici* J9 in a mice model: intake of nutrition and water

There was no significant change in the control group and the experimental material administration group in the amount of intake of feed and water during the experiment period (Table 3). Although the water consumption was significantly reduced on day 14 when compared to day 7, since it was both seen in the control group and the administration group, so it could be regarded as the environmental effect, not an effect of administration. Therefore, it seems that the administration of experimental materials does not affect significantly the amount of intake of feed and water (Fig. 1b and c).

**Table 3:** Daily food and water consumption of male and female (mice) treated orally with *P. acidilactici* J9 for 14 days

Sex	Dose (mg)	Food consumption (g/day/mouse)		Water consumption (mL/day/mouse)	
		Days after treatment		Days after treatment	
		7	14	7	14
Male	Control	74^NS^	87^NS^	139^NS^	120^NS^
	500	92	91	141	118
	1000	71	95	137	111
	2000	113	93	154	137
Female	Control	103^NS^	83^NS^	127^NS^	124^NS^
	500	85	81	148	129
	1000	108	79	142	152
	2000	65	70	132	112

Values are expressed as mean (*n* = 5)

*NS* not significantly different among groups

### Oral toxicity study of *P. acidilactici* J9 in a mice model: necropsy results

As the result of the observation of main organs with naked eyes after the necropsy of experimental mice, there was no significant change in organs and specific necropsy opinion dependent on the dose of administration (Table 4). Some mice from female control group were excluded from the data due to black damaged areas of the spleen during organ harvest, which is just the characteristics of a mouse individual. However in both control group and administration group, blackish red discoloration at the spleen terminal, shrinkage of the right testicle, and thinning of the right atrium were observed. (CTR-F-001: discoloration of spleen; CTR-F-005: discoloration of spleen; 500 mg-F-001: discoloration of spleen; CTR-M-004: discoloration of spleen; CTR-M-005: thinning of right atrium; 500 mg-M-004: discoloration of spleen; 1000 mg-M-004: shrinkage of right testicle) However, as the same phenomenon was seen in the control group and the administration group, these are the characteristics of a mouse individual and is not an effect of administration.

**Table 4:** Gross findings of male and female (mice) treated orally with *P. acidilactici* J9 for 14 days

Sex		Male				Female			
Dose (mg)		Control	500	1000	2000	Control	500	1000	2000
Brain	NGF	5(100)	5(100)	5(100)	5(100)	5(100)	5(100)	5(100)	5(100)
Lung	NGF	5(100)	5(100)	5(100)	5(100)	5(100)	5(100)	5(100)	5(100)
Liver	NGF	5(100)	5(100)	5(100)	5(100)	5(100)	5(100)	5(100)	5(100)
Heart	NGF	4(90)	5(100)	5(100)	5(100)	5(100)	5(100)	5(100)	5(100)
Kidney(L)	NGF	5(100)	5(100)	5(100)	5(100)	5(100)	5(100)	5(100)	5(100)
Kidney(R)	NGF	5(100)	5(100)	5(100)	5(100)	5(100)	5(100)	5(100)	5(100)
Testis(L)	NGF	5(100)	5(100)	5(100)	5(100)				
Testis(R)	NGF	5(100)	5(100)	4(90)	5(100)				
Ovary(L)	NGF					5(100)	5(100)	5(100)	5(100)
Ovary(R)	NGF					5(100)	5(100)	5(100)	5(100)
Spleen	NGF	4(90)	4(90)	5(100)	5(100)	3(70)	4(90)	5(100)	5(100)
Thymus	NGF	5(100)	5(100)	5(100)	5(100)	5(100)	5(100)	5(100)	5(100)

Values are expressed as animal numbers (the percentage of animal numbers)

*NGF* no gross finding

### Oral toxicity study of *P. acidilactici* J9 in a mice model: the weight of organs

The weight of organs were measured after repeated administration of *P. acidilactici* J9 which varied to low dose (500 mg/kg/day), medium dose (1000 mg/kg/day), and high dose (2000 mg/kg/day) for 2 weeks (Table 5). No changes were observed in the weight of brain, lung, testis, ovary, kidney, heart, spleen, and liver with respect to the administration of experimental materials and no abnormal changes were dependent on dose of administrations. Generally, when the toxic materials were ingested, liver takes the largest effect since the detoxification starts at the liver. However, there were no significant changes in each group on the observed weight of the liver. From the results above, the administration of *P. acidilactici* J9 does not affect the weight of organs.

**Table 5:** Organ weights of male and female (mice) treated orally with *P. acidilactici* J9 for 14 days

		(Unit: g)			
Sex	Organs	Dose (mg)			
		Control	500	1000	2000
Male	Brain	0.445 ± 0.014	0.440 ± 0.014	0.444 ± 0.015	0.437 ± 0.016
	Lung	0.124 ± 0.013	0.126 ± 0.006	0.125 ± 0.010	0.134 ± 0.023
	Liver	0.984 ± 0.208	0.920 ± 0.065	0.929 ± 0.183	0.904 ± 0.176
	Heart	0.104 ± 0.011	0.101 ± 0.007	0.100 ± 0.008	0.105 ± 0.010
	Kidney(L)	0.129 ± 0.025	0.128 ± 0.025	0.127 ± 0.13	0.126 ± 0.018
	Kidney(R)	0.133 ± 0.025	0.136 ± 0.011	0.134 ± 0.017	0.140 ± 0.029
	Testis(L)	0.071 ± 0.020	0.067 ± 0.000	0.067 ± 0.008	0.071 ± 0.006
	Testis(R)	0.074 ± 0.024	0.070 ± 0.000	0.063 ± 0.018	0.074 ± 0.007
	Spleen	0.057 ± 0.009	0.052 ± 0.007	0.050 ± 0.006	0.054 ± 0.014
	Thymus	0.042 ± 0.010	0.046 ± 0.007	0.044 ± 0.007	0.045 ± 0.008
Female	Brain	0.443 ± 0.014	0.434 ± 0.019	0.437 ± 0.013	0.431 ± 0.018
	Lung	0.120 ± 0.006	0.120 ± 0.008	0.134 ± 0.014	0.121 ± 0.017
	Liver	0.775 ± 0.065	0.788 ± 0.199	0.755 ± 0.092	0.727 ± 0.103
	Heart	0.091 ± 0.007	0.089 ± 0.005	0.105 ± 0.007	0.084 ± 0.007
	Kidney(L)	0.114 ± 0.025	0.102 ± 0.011	0.126 ± 0.007	0.102 ± 0.010
	Kidney(R)	0.110 ± 0.011	0.110 ± 0.013	0.140 ± 0.012	0.105 ± 0.012
	Ovary (L)	0.001 ± 0.000	0.002 ± 0.000	0.002 ± 0.000	0.001 ± 0.000
	Ovary(R)	0.002 ± 0.000	0.002 ± 0.000	0.002 ± 0.000	0.002 ± 0.000
	Spleen	0.058 ± 0.007	0.052 ± 0.010	0.053 ± 0.010	0.051 ± 0.007
	Thymus	0.077 ± 0.007	0.071 ± 0.010	0.045 ± 0.011	0.076 ± 0.014

Values are expressed as mean ± SE (*n* = 5)

*NS* not significantly different among groups

### Oral toxicity study of *P. acidilactici* J9 in a mice model: hematological tests

As the result of the measurement of hematological parameters, no significant changes were observed in control and administration groups (*p* ≤ 0.05) (Table 6). As a result of hematological examination, both the control group and the administration group were included in normal range and no dependence on dose was observed. This result is similar to the range previously reported in hematological fundamental database of which there is a repeated toxicity test for 2 weeks using mice.

**Table 6:** Hematology of male and female (mice) treated orally with *P. acidilactici* J9 for 14 days

Sex	Parameters		Dose (mg)			
			Control	500	1000	2000
Male	CBC	WBC (×10^3^/μL)	2.672 ± 0.65	2.066 ± 0.63	2.016 ± 1.01	2.474 ± 0.96
		RBC (×10^6^/μL)	9.88 ± 0.33	9.518 ± 0.26	10.00 ± 0.14	9.876 ± 0.32
		HGB (g/dL)	15.14 ± 0.64	14.72 ± 0.36	15.36 ± 0.28	15.26 ± 0.53
		HCT (%)	51.62 ± 2.24	49.42 ± 1.43	51.38 ± 1.17	50.90 ± 2.09
		MCV (fL)	52.26 ± 0.69	51.9 ± 0.42	51.36 ± 1.09	51.54 ± 0.72
		MCH (pg)	15.32 ± 0.22	15.46 ± 0.21	15.34 ± 0.22	15.46 ± 0.23
		MCHC (g/dL)	29.30 ± 0.23	29.78 ± 0.29	29.88 ± 0.43	29.96 ± 0.40
		CHCM (g/dL)	28.16 ± 0.35	28.16 ± 0.11	28.64 ± 0.44	28.68 ± 0.45
		RDW (%)	13.36 ± 0.38	13.88 ± 0.40	13.30 ± 0.50	13.26 ± 0.94
		HDW (g/dL)	1.45 ± 0.02	1.45 ± 0.06	1.45 ± 0.04	1.45 ± 0.03
		CH (pg)	14.68 ± 0.19	14.58 ± 0.08	14.66 ± 0.17	14.74 ± 0.09
		CHDW (%)	2.00 ± 0.05	2.07 ± 0.08	2.00 ± 0.05	2.01 ± 0.11
		PLT (×10^3^/μL)	1263.20 ± 73.52	1244.80 ± 57.87	1238.60 ± 61.75	1202.00 ± 60.76
		MPV (fL)	7.44 ± 0.17	7.88 ± 0.05	7.52 ± 0.08	7.48 ± 0.26
		PDW (%)	60.38 ± 1.85	57.30 ± 1.62	55.80 ± 1.62	55.96 ± 2.34
		PCT (%)	0.94 ± 0.06	0.98 ± 0.05	0.93 ± 0.05	0.90 ± 0.06
	DIFF	NEUT (×10^3^/μL)	0.34 ± 0.13	0.18 ± 0.05	0.19 ± 0.09	0.21 ± 0.05
		NEUT (%)	12.5 ± 3.19	9.26 ± 2.15	9.56 ± 1.06	8.94 ± 1.50
		LYMPH (×10^3^/μL)	2.25 ± 0.52	1.83 ± 0.56	1.78 ± 0.89	2.22 ± 0.89
		LYMPH (%)	84.46 ± 3.46	88.48 ± 2.27	88.52 ± 1.21	89.46 ± 1.17
		MONO (×10^3^/μL)	0.02 ± 0.01	0.01 ± 0.01	0.01 ± 0.01	0.01 ± 0.01
		MONO (%)	0.54 ± 0.34	0.32 ± 0.28	0.28 ± 0.18	0.32 ± 0.16
		EOS (×10^3^/μL)	0.05 ± 0.02	0.03 ± 0.04	0.02 ± 0.01	0.01 ± 0.01
		EOS (%)	1.64 ± 0.50	1.30 ± 1.31	0.76 ± 0.30	0.48 ± 0.21
		BASO (×10^3^/μL)	0.01 ± 0.01	0.01 ± 0.01	0.01 ± 0.01	0.01 ± 0.01
		BASO (%)	0.28 ± 0.13	0.40 ± 0.20	0.34 ± 0.24	0.26 ± 0.19
		LUC (×10^3^/μL)	0.02 ± 0.01	0.01 ± 0.01	0.01 ± 0.01	0.02 ± 0.01
		LUC (%)	0.62 ± 0.16	0.24 ± 0.27	0.48 ± 0.33	0.56 ± 0.23
	RETI	Reticulocyte (×10^9^/μL)	301.60 ± 47.96	258.32 ± 30.72	302.89 ± 10.26	298.02 ± 63.69
		Reticulocyte (%)	3.15 ± 0.51	2.71 ± 0.25	3.03 ± 0.12	3.00 ± 0.56
		MCVr (fL)	58.00 ± 0.51	57.46 ± 0.48	57.60 ± 0.65	57.86 ± 1.13
		CHCMr (g/dL)	26.24 ± 0.17	25.92 ± 0.13	26.30 ± 0.19	26.18 ± 0.31
		RDWr (%)	12.06 ± 0.65	12.36 ± 0.66	11.96 ± 0.64	12.20 ± 0.85
		HDWr (%)	2.34 ± 0.12	2.40 ± 0.10	2.43 ± 0.07	2.52 ± 0.10
		CHr (pg)	15.20 ± 0.25	14.86 ± 0.22	15.12 ± 0.11	15.12 ± 0.16
		CHDWr (%)	1.98 ± 0.08	1.97 ± 0.07	1.92 ± 0.05	1.97 ± 0.06
Female	CBC	WBC (×10^3^/μL)	2.95 ± 0.60	2.53 ± 1.08	2.75 ± 0.70	3.32 ± 1.34
		RBC (×10^6^/μL)	9.51 ± 0.13	10.19 ± 0.15	10.07 ± 0.22	9.97 ± 0.31
		HGB (g/dL)	14.72 ± 0.21	15.80 ± 0.47	15.60 ± 0.26	15.74 ± 0.31
		HCT (%)	49.12 ± 0.73	52.20 ± 1.70	51.48 ± 1.52	50.44 ± 1.77
		MCV (fL)	51.64 ± 0.43	51.16 ± 1.04	51.12 ± 0.79	50.60 ± 0.62
		MCH (pg)	15.50 ± 0.23	15.48 ± 0.33	15.50 ± 0.33	15.78 ± 0.42
		MCHC (g/dL)	29.98 ± 0.36	30.28 ± 0.35	30.32 ± 0.61	31.20 ± 0.91
		CHCM (g/dL)	28.74 ± 0.34	28.94 ± 0.40	28.96 ± 0.43	29.5 ± 0.35
		RDW (%)	13.68 ± 0.46	13.90 ± 0.46	13.00 ± 0.52	13.02 ± 0.46
		HDW (g/dL)	1.50 ± 0.05	1.54 ± 0.03	1.52 ± 0.04	1.59 ± 0.02
		CH (pg)	14.76 ± 0.13	14.74 ± 0.21	14.76 ± 0.15	14.86 ± 0.06
		CHDW (%)	2.07 ± 0.06	2.11 ± 0.05	2.00 ± 0.08	2.03 ± 0.06
		PLT (×10^3^/μL)	955.60 ± 64.27	1102.40 ± 63.27	1129.80 ± 94.82	1035.00 ± 107.62
		MPV (fL)	7.56 ± 0.35	7.70 ± 0.16	7.82 ± 0.11	7.38 ± 0.47
		PDW (%)	57.78 ± 3.04	57.10 ± 2.82	54.94 ± 1.31	57.68 ± 3.19
		PCT (%)	0.73 ± 0.07	0.85 ± 0.06	0.89 ± 0.08	0.77 ± 0.11
	DIFF	NEUT (×10^3^/μL)	0.28 ± 0.07	0.29 ± 0.06	0.25 ± 0.08	0.23 ± 0.12
		NEUT (%)	9.48 ± 0.42	12.38 ± 2.50	9.70 ± 3.01	6.94 ± 1.34
		LYMPH (×10^3^/μL)	2.61 ± 0.53	2.20 ± 1.01	2.45 ± 0.72	3.03 ± 1.19
		LYMPH (%)	88.50 ± 0.39	86.24 ± 2.40	88.74 ± 3.30	91.42 ± 1.61
		MONO (×10^3^/μL)	0.01 ± 0.00	0.00 ± 0.01	0.01 ± 0.01	0.01 ± 0.00
		MONO (%)	0.46 ± 0.15	0.20 ± 0.12	0.22 ± 0.13	0.22 ± 0.08
		EOS (×10^3^/μL)	0.02 ± 0.01	0.01 ± 0.01	0.01 ± 0.01	0.01 ± 0.01
		EOS (%)	0.80 ± 0.25	0.34 ± 0.38	0.34 ± 0.26	0.42 ± 0.29
		BASO (×10^3^/μL)	0.01 ± 0.01	0.01 ± 0.01	0.01 ± 0.00	0.01 ± 0.01
		BASO (%)	0.32 ± 0.22	0.30 ± 0.12	0.38 ± 0.19	0.30 ± 0.19
		LUC (×10^3^/μL)	0.01 ± 0.01	0.02 ± 0.01	0.02 ± 0.01	0.03 ± 0.02
		LUC (%)	0.48 ± 0.13	0.54 ± 0.18	0.64 ± 0.51	0.66 ± 0.38
	RETI	Reticulocyte (×10^9^/μL)	309.34 ± 52.93	304.60 ± 43.32	277.50 ± 38.28	351.10 ± 49.29
		Reticulocyte (%)	3.25 ± 0.53	2.99 ± 0.42	2.76 ± 0.41	3.53 ± 0.60
		MCVr (fL)	57.90 ± 0.50	57.86 ± 1.25	57.36 ± 0.70	58.12 ± 0.75
		CHCMr (g/dL)	26.46 ± 0.18	26.42 ± 0.33	26.44 ± 0.11	26.76 ± 0.23
		RDWr (%)	13.08 ± 0.93	12.14 ± 1.01	13.40 ± 0.70	13.22 ± 1.00
		HDWr (%)	2.58 ± 0.11	2.63 ± 0.11	2.75 ± 0.12	2.81 ± 0.12
		CHr (pg)	15.30 ± 0.23	15.24 ± 0.42	15.12 ± 0.22	15.52 ± 0.13
		CHDWr (%)	2.03 ± 0.04	2.01 ± 0.08	2.07 ± 0.07	2.13 ± 0.10

Values are expressed as mean ± SE (*n* = 5)

*NS* not significantly different among groups

### Oral toxicity study of *P. acidilactici* J9 in a mice model: blood biochemical analysis

As the result of the measurement of the indicator of blood biochemistry, no significant changes dependent on the administration of experimental materials were observed in the whole administration groups with respect to the control group. Both the control group and *P. acidilactici* J9 administration group showed normal parameters (*p* ≤ 0.05) (Table 7).

**Table 7:** Levels of serum biochemical indices of male and female (mice) treated orally with *P. acidilactici* J9 for 14 days

Sex	Parameters	Dose (mg)			
		Control	500	1000	2000
Male	Glu	256.40 ± 11.91	243.00 ± 42.57	234.20 ± 25.82	230.80 ± 31.32
	BUN	18.68 ± 3.87	21.54 ± 5.78	20.50 ± 6.22	18.24 ± 2.60
	Crea	0.28 ± 0.04	0.29 ± 0.02	0.27 ± 0.02	0.27 ± 0.03
	T-Chol	79.00 ± 4.36	85.20 ± 4.92	82.40 ± 6.80	81.80 ± 4.66
	TP	4.74 ± 0.05	4.82 ± 0.24	4.76 ± 0.11	4.68 ± 0.16
	ALB	1.70 ± 0.07	1.72 ± 0.08	1.68 ± 0.04	1.68 ± 0.13
	T-BIL	0.06 ± 0.05	0.04 ± 0.05	0.08 ± 0.04	0.08 ± 0.04
	ALP	133.00 ± 19.51	137.80 ± 9.65	126.20 ± 19.82	137.40 ± 15.96
	AST (GOT)	56.20 ± 15.07	65.20 ± 13.18	48.00 ± 7.35	57.40 ± 13.28
	ALT (GPT)	28.80 ± 4.15	28.00 ± 4.18	22.20 ± 1.30	25.20 ± 4.49
	TG	66.40 ± 26.88	64.40 ± 31.45	49.80 ± 23.85	39.40 ± 17.94
	A/G	0.56 ± 0.05	0.56 ± 0.05	0.52 ± 0.04	0.56 ± 0.05
	B/C	66.61 ± 12.59	74.13 ± 18.80	76.83 ± 22.46	69.05 ± 11.10
Female	Glu	214.40 ± 37.40	229.60 ± 47.45	228.40 ± 40.32	231.00 ± 66.87
	BUN	25.16 ± 5.42	24.70 ± 5.07	23.06 ± 3.26	22.58 ± 4.50
	Crea	0.27 ± 0.05	0.25 ± 0.05	0.28 ± 0.03	0.26 ± 0.03
	T-Chol	76.80 ± 8.11	70.20 ± 5.97	78.80 ± 13.44	80.00 ± 9.85
	TP	4.82 ± 0.08	4.72 ± 0.16	4.78 ± 0.20	4.70 ± 0.23
	ALB	1.74 ± 0.05	1.74 ± 0.05	1.74 ± 0.05	1.72 ± 0.04
	T-BIL	0.00 ± 0.00	0.02 ± 0.04	0.02 ± 0.04	0.02 ± 0.04
	ALP	168.20 ± 8.93	173.40 ± 10.31	154.20 ± 13.88	153.60 ± 26.37
	AST (GOT)	68.20 ± 17.02	67.60 ± 14.57	66.80 ± 8.20	78.40 ± 18.01
	ALT (GPT)	21.00 ± 12.88	22.80 ± 3.11	24.60 ± 3.51	23.00 ± 3.39
	TG	0.56 ± 0.05	43.00 ± 9.70	27.00 ± 3.00	34.20 ± 15.45
	A/G	0.56 ± 0.05	0.58 ± 0.04	0.56 ± 0.05	0.58 ± 0.04
	B/C	94.97 ± 12.02	103.82 ± 32.56	82.32 ± 13.29	87.08 ± 24.74

Values are expressed as mean ± SE (*n* = 5)

*NS* not significantly different among groups

### Oral toxicity study of *P. acidilactici* J9 in a mice model: histopathology observations

**Fig. 2:**
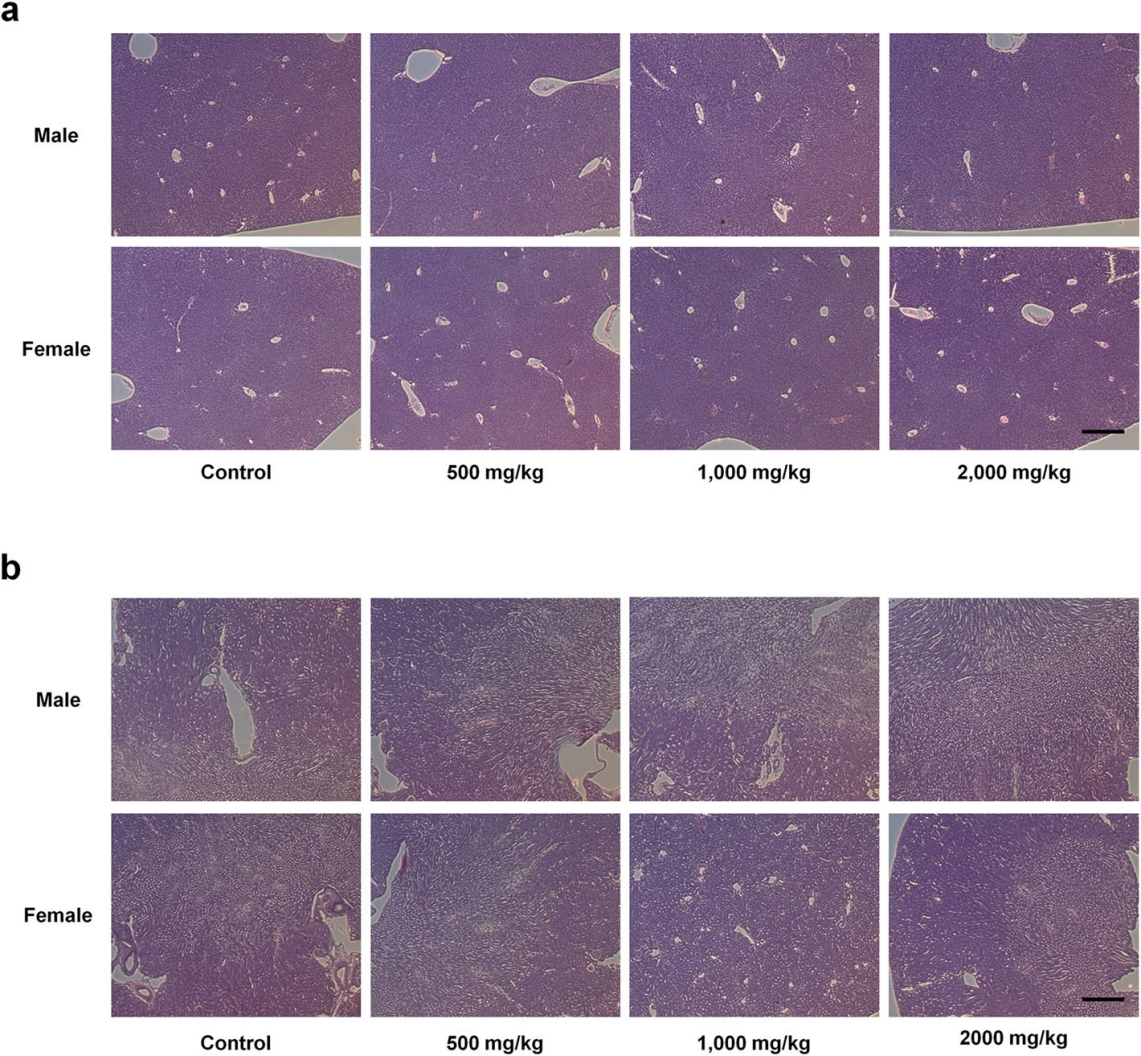
Histopathological examinations of the liver and Kidney. **a, b** Female and male C57BL / 6 mice were orally administered with *P. acidilactici* J9 for 14 days. The liver and kidneys of the control and *P. acidilactici* J9 administration animals were extracted and fixed with 10% neutral buffered formalin solution on the final necropsy day of all animals after gross lesion observation. Pathological lesions and structures such as liver, kidney infection, necrosis, and iron and bilirubin pigmentation were confirmed by H & E staining. Bar = 30 μm, *N* = 10 samples per group

For the histopathology test of *P. acidilactici* J9-administrated mice, liver and kidney were stained by hematoxylin and eosin. We have chosen to observe liver and kidney because those two are representative organs that react to toxicity [27]. As the result of the histopathology test, no lesions were observed in the liver, like infection, necrosis, iron pigmentation, and bilirubin pigmentation. The structure of liver cells were also normal (Fig. 2a). There was no lesions in the kidney, like infection and necrosis, and no changes were observed in kidney cells (Fig. 2b). Therefore, there is no significant changes in liver and kidney, and no extraordinary pathologic abnormality dependent on dose of experimental materials were observed in both the control group and administration group as the result of the histopathology test. This opinion seems to correspond with the long-term change of weight as well as the blood biochemical change. Mijung Lee had validated the histopathology findings.

### Inhibition of adhesion and growth of *H.pylori* in gastric epithelial cells in the presence of *P. acidilactici* J9

**Fig. 3:**
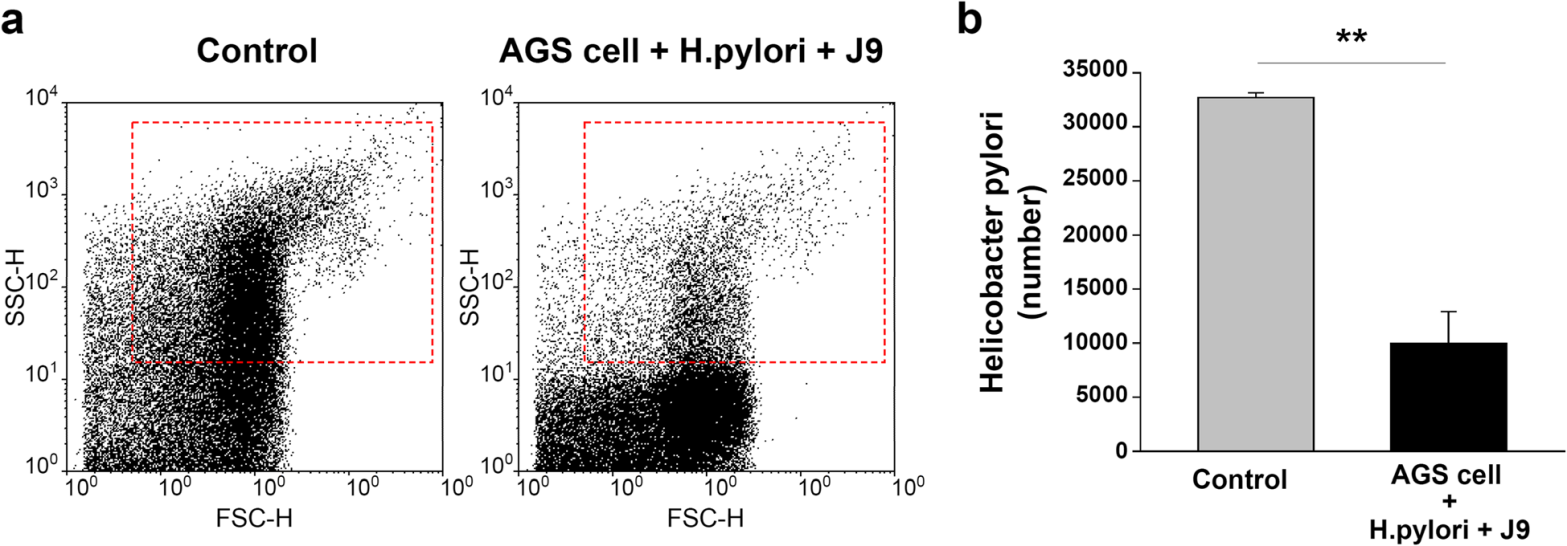
*P. acidilactici* J9 inhibits adhesion and growth of *H. pylori* in gastric epithelial cells. **a** After *H. pylori* supernatant and *P. acidilactici* J9 were treated for 24 h in AGS cells, *H. pylori* count was confirmed by flow cytometry. In the control group, the *H.pylori* number was confirmed by flow measurement after 24 h of treatment with *H.pylori* in the AGS cell. **b** *H. pylori* number was quantified by a flow cytometer. The experiment was repeated three times and the data are shown as the mean (SEM). **p < 0.01 versus control group

The adhesion and growth of *H. pylori* were inhibited by a 24 h treatment of *H. pylori* and *P. acidilactici* J9 on AGS cells, which are gastric cancer cells. Compared to the control group (AGS cell and H.pylori), the number of *H. pylori* analyzed by FACS significantly (*p* < 0.01) decreased after incubation of AGS cell with *P. acidilactici* J9 for 24 h. Control biological triplicate groups are also analyzed for statistical options (Fig. 3 and Figure S1 in Additional file 1).

## Discussion

*P. acidilactici* J9 exerts as an antagonism against other enteric pathogens, primarily through the production of lactic acid and secretion of bacteriocin [28–34]. Thermo-stable bacteriocin is an antimicrobial peptide known to have a strong activity against food bacteria and pathogenic enteric bacteria [28, 35–38]. For these reasons, bacteriocin secreted by *P. acidilactici* J9 has a potential to inhibit other pathogenic bacteria, and probiotics including *P. acidilactici* J9 have potential to be used for commercial healthcare products like beverages and foods [21]. However, a systematic study on its repeated oral administration toxicity has not been reported yet.

Various antibiotics have been used for *H.pylori* eradication [39–42], since it is known to be an important causative agent of peptic ulcer, gastritis, gastric cancer, or mucosa associated lymphoid tissue lymphoma [43]. These antimicrobial agents have been pointed out for various problems such as adverse effects, risk of re-infection due to increased pH, appearance of resistant bacteria, and high cost [42, 44–47]. Recently, there is growing interest in probiotic lactic acid bacteria, which can play a role in the treatment of *H.pylori* by directly acting on *H.pylori*, with minimal clinical side effects of antibiotics [48–52].

This study investigated the toxicity and anti-*H.pylori* effect of *P. acidilactici* J9. Daily administration of *P. acidilactici* J9 in mice for 2 week showed no abnormal clinical signs in body weight, hematology, food intake and water consumption. In all test groups, no general symptoms and deaths from the test substance were observed. During the entire test period, body weight continuously increased but no significant change was observed with the control group. In addition, there were no significant differences in the gross observation, long-term weight change, hematology, blood biochemical and histopathologic examination of the organ in all the test substance administration groups, and all of them were within the normal range. As a result of repeated toxicity test for 2 weeks, *P. acidilactici* J9 was judged to be a safe and low-toxic substance. But further investigation will be needed to interpret the data that female mice at 1000 mg of dose showed higher heart, kidney (L, R) weight and lower thymus weight when compared to control, and that female mice at 500 and 2000 mg of dose showed lower kidney (L) weight when compared to control.

*P. acidilactici* J9, inhibits the adhesion of *H.pylori* to AGS gastric cancer cells. Probiotics refers to living microorganisms that are beneficial to the human body when consumed in moderate quantities [53, 54]. Most probiotics known to date are lactic acid bacteria [55, 56]. Probiotic bacteria such as lactic acid bacteria and beneficial bacteria survive in the stomach acid and bile acid in the body, reach the small intestine, multiply in the intestines and settle [57, 58]. It has a beneficial effect on health in the colon, and these probiotics should be non-toxic and non-pathogenic [59, 60]. Ingestion of probiotics not only helps maintain health, it also helps to improve various diseases such as infants, irritable bowel syndrome, and inflammatory bowel disease [61].

Based on our in vivo and in vitro results, when used as probiotics, *P. acidilactici* J9 may help decrease the occurrence of gastritis and reduce the risk of *H.pylori* infection with promising safety issues, without side effects.

## Conclusions

In conclusion, we reported the toxicity and anti-*H.pylori* effect of *P. acidilactici* J9. Daily administration of *P. acidilactici* J9 in mice for 2 week showed no abnormal clinical signs in body weight, hematology, food intake and water consumption. Also, *P. acidilactici* J9, inhibited the adhesion of *H.pylori* to AGS gastric cancer cells. Based on our in vivo and in vitro results, when used as probiotics, *P. acidilactici* J9 might have the potential to decrease the occurrence of gastritis and the risk of *H.pylori* infection with promising safety issues, without side effects.

## Methods

### Model organisms and conditions

C57BL/6 mice of 4 weeks of age without certain pathogens are purchased from Orient Bio (Seongnam, Korea) at an amount of 20 males and females each. Normal and healthy mice without any weight loss are used in experiment by clinical observation during 7 days of education. Feeds are the following; solid feeds for laboratory animal are freely offered, and drinking water. The filtration-purified water is also freely offered to mice. The mice were house in groups with ad libitum access to food and water and a 12 h light / 12 h dark cycle. Also, *P. acidilactici* J9 has been prepared through Industry Promotion Administration.

### Configuration of test group and set of dosage setting

Dosage was set by MFDS (Ministry of Food and Drug Safety) standards. Maximum dosage is set to 2000 mg/kg/day for both male and female, with the geometric ratio of 1/2, low dose group, medium dose group, and high dose group are set at 500, 1000, and 2000 mg per body weight (kg) respectively. 500, 1000, 2000 mg of dosage corresponds to 5 × 10^8^ CFU, 1 × 10^9^ CFU, and 2 × 10^9^ CFU, however, we used the mg/ml unit which is more commonly used in the animal experiments. The number of mice in each group are set to 5 males and females each. Dosage is set to not exceed 0.2 ml per 10 g and calculated according to the body weight measured just before administration. Test materials are well-mixed to sterile distilled water before administration, and they are directly injected into the stomach by sonde for oral administration for once a day during 2 weeks. Sterile distilled water for injection is used as reference material.

### Normal symptoms and observation of lethality in mice

Observation is conducted for 6 h after oral administration and starting from the next day, to observe change of general condition, as well as expression of addiction. This was held in presence of dead mice and symptoms that can be expressed by the test materials are observed carefully. In the case of abnormality, the type and the extent of symptoms are recorded individually. All mice were checked for death or critical condition.

### Measurement of weight, feed and water intake

For every animal, change of weight is measured just before the administration of test materials once a week at a certain time during 2 weeks. Intake of feeds and water is measured and calculated weekly.

### Necropsy and naked eye examination

Mice were anesthetized by CO_2_; the air atmosphere of chamber that contains mice was replaced to CO_2_ with the volume displacement rate of 20%/min, and all surgical procedures were performed under general anesthesia. Euthanasia of mice was done by collecting 0.5 ml – 0.8 ml of blood from the heart under anesthesia. The protocols were in accordance with official governmental guidelines, and all efforts were made to minimize the number of mice used and their suffering. Also, other organs was obtained by mice. The brain, kidney, liver, lung, reproductive organ, heart, spleen, and thymus are extracted and weighed. External findings such as abnormality of subcutaneous, internal organs and brain were observed with the naked eye.

### Blood biochemical

The hematologic analysis of the serum is performed the same day of the necropsy, which is collected from a 3000 rpm, 20 min long, centrifugation of the blood and conducted by auto-analyzer (Hitachi-747, Hitachi Medical Co., Tokyo, Japan). Glucose; GLU, Blood Urea Nitrogen; BUN, Creatinine; CREA, Total cholesterol; T-CHOL, Albumin; ALB, *Total Bilirubin; T-BIL,* Alkaline Phosphatase; ALP, Aspartate Aminotransferase; AST (GOT), Alanine Aminotransferase; ALT (GPT), Triglyceride; TG, and Total protein; TP are measured.

### Hematology

Mice were fasted for 8 h followed by the anesthetization before the necropsy. Part of the blood from the exsanguination is EDTA-treated and stored in tubes and then analyzed by blood auto-analyzer (System SE-9000, TOAMedical Electronics Co., Ltd., Kobe, Japan). Red blood cells, RBC, hematocrit, HCT, hemoglobin, Hb, mean corpuscular volume, MCV, mean corpuscular hemoglobin, MCH, mean corpuscular hemoglobin concentration, MCHC, white blood cells, WBC, Hemoglobin, HGB, Cellular Hb Concentration Mean, CHCM, Red Cell Distribution Width, RDW, Hb Distribution Width, HDW, Cellular Hb content, CH, Cellular Hb Distribution Width, CHDW, Platelet, PLT, Platelet Distribution Width, PDW, Plateletcrit, PCT, Neutrophil, NEUT, Neutrophil, NEUT%, Lymphocyte, LYMPH, Lymphocyte %, LYMPH%, Monocyte, MONO, Monocyte %, MONO%, Eosinophil, EOS, Eosinophil %, EOS%, Basophil, BASO, Basophil %, BASO%, Large Unstained Cells, LUC, Large Unstained Cells, LUC%, Reticulocyte Count, Retic#, Reticulocyte %, Retic%, Mean Corpuscular Volume of Retics, MCVr, Mean Corpuscular Volume of Retics %, MCVr%, Red Cell Distribution Width of Retics, RDWr*, Hb Distribution Width of Retics, HDWr*, Cellular Hb of Retics, CHr, and Cellular Hb Distribution Width of Retics, CHDWr* are measured.

### Histopathology

Liver and kidney were extracted and fixed with a 10% neutral buffered formalin solution the day of final necropsy, after the observation of gross lesions on every animal which were administered with test materials. Then paraffin embedding wass conducted and hematoxylin & eosin dye performed with the sections of 3 ~ 4 um sections. Mijung Lee had validated the findings.

### *H. pylori* preparation

*H. pylori* (ATCC 43504) used in this study were obtained and inoculated onto chocolate media, incubated for 5 ~ 7 days at 37 °C in a 10% CO_2_ incubator under aerobic conditions and then used for the examination. When the chocolate media is filled over 90%, *H. pylori* is swabbed with sterilized swabs and suspended in 20 ml of RPMI-1640 media to form the *H. pylori* suspension.

### Cell culture

The human gastric adenocarcinoma cell lines AGS (KCLB 21739; Korea) cells were seeded at a density of 1 × 10^5^ cells in 2 ml of RPMI-1640 (RPMI-1640; Gibco, Carlsbad, CA, USA) supplemented with 10% fetal bovine serum (FBS; Gibco, Carlsbad, CA, USA) and 1% penicillin-streptomycin (Invitrogen, USA) into 6 well culture plates (SPL) and cultured for 2 ~ 3 days at 37 °C in a 5% CO_2_ incubator.

### Adhesion assay

When the AGS cell reach a density of 80% of the seeding plate, we eliminate the media from the plate and wash with phosphate buffered saline (PBS: Welgene, Daegu, Korea) 3 times. Experimental groups are as follows. For negative control, only AGS is seeded. For positive control, AGS is treated by 1 ml of *H. pylori* suspension. For the measurement of suppression of attachment, AGS is treated by 1 ml of *H. pylori* suspension (1 × 10^8^ CFU/ml) and *P. acidilactici* J9 (1 × 10^8^ CFU/ml) at a multiplicity of infection of 100. The culture plates seeded with AGS treated by *H. pylori* and *P. acidilactici* J9 are incubated for 90 min at 37 °C in a 5% CO_2_ incubator. The culture media is eliminated and the cells are carefully harvested. The cells are suspended in 500ul of PBS then examined with FACS.

### Flow cytometry

The culture media is eliminated and the cells were washed and carefully harvested in PBS (phosphate buffered saline, WelGene, Daegu, Korea) using a cell scraper. Cells were counted and 1 × 10^6^ cells were suspended in 1 ml cold PBS. Cells (5 × 10^6^ or 1 × 10^7^) were centrifuged at 1200 rpm for 5 min. The cells are suspended in 500ul of PBS then flow cytometric analysis was performed (FACS Calibur, BD Bioscience, CA, USA). The data were analyzed using Flowing Software (www.flowingsoftware.com).

### Statistical analysis

All values shown in the figures are presented as mean ± standard error. A 2-tailed probability value below 0.05 was considered statistically significant. Data were analyzed using SPSS version 17.0 (SPSS Inc., USA).

## Supplementary Information

**Additional file 1. **Populations of *H.pylori* were analyzed with a flow cytometer. When the chocolate media was filled with over 90%, *Helicobacter pylori* was swabbed with sterilized swabs and suspended in RPMI-1640 media 20 ml to form *Helicobacter pylori* suspension. The population in a red gate were assayed as *Helicobacter pylori*.

**Additional file 2.**

## Data Availability

Data generated by and used in the study is available from the corresponding author upon reasonable request.

## Abbreviations

*P. acidilactici*: *Pediococcus acidilactici*
*H. pylori*: *Helicobacter pylori*
AGS: Adenocarcinoma gastric
LAB: Lactic acid bacteria
RBC: Red blood cells
HCT: Hematocrit
Hb: Hemoglobin
MCV: Mean corpuscular volume
MCH: Mean corpuscular hemoglobin
MCHC: Mean corpuscular hemoglobin concentration
WBC: White blood cells
HGB: Hemoglobin
CHCM: Cellular Hb Concentration Mean
RDW: Red Cell Distribution Width
HDW: Hb Distribution Width
CH: Cellular Hb content
CHDW: Cellular Hb Distribution Width
PLT: Platelet
PDW: Platelet Distribution Width
PCT: Plateletcrit
NEUT: Neutrophil
NEUT%: Neutrophil %
LYMPH: Lymphocyte
LYMPH%: Lymphocyte %
MONO: Monocyte
MONO%: Monocyte %
EOS: Eosinophil
EOS%: Eosinophil %
BASO: Basophil
BASO%: Basophil %
LUC: Large Unstained Cells
LUC%: Large Unstained Cells
Retic#: Reticulocyte Count
Retic%: Reticulocyte %
MCVr: Mean Corpuscular Volume of Retics
MCVr%: Mean Corpuscular Volume of Retics %
RDWr*: Red Cell Distribution Width of Retics
HDWr*: Hb Distribution Width of Retics
CHr: Cellular Hb of Retics
CHDWr*: Cellular Hb Distribution Width of Retics
GLU: Glucose
BUN: Blood Urea Nitrogen
CREA: Creatinine
T-CHOL: Total cholesterol
ALB: Albumin
*T-BIL*: *Total Bilirubin*
ALP: Alkaline Phosphatase
AST (GOT): Aspartate Aminotransferase
ALT (GPT): Alanine Aminotransferase
TG: Triglyceride
TP: Total protein
FACS: Fluorescence-activated cell sorting
