# Supplementary material for: Two-weeks repeated-dose oral toxicity study of Pediococcus acidilactici J9 in a mice model
Source: BMC Microbiol. 2020 Dec 9;20:372. doi: 10.1186/s12866-020-02055-4 (PMC7727177; doi:10.1186/s12866-020-02055-4)
Supplement: Supplementary file 1 — Additional file 1. Populations of H.pylori were analyzed with a flow cytometer. When the chocolate media was filled with over 90%, Helicobacter pylori was swabbed with sterilized swabs and suspended in RPMI-1640 media 20 ml to form Helicobacter pylori suspension. The population in a red gate were assayed as Helicobacter pylori. [file 12866_2020_2055_MOESM1_ESM.docx]

**Two-weeks Repeated-dose Oral Toxicity Study of *Pediococcus acidilactici* J9 in a Mice model**

Mijung Lee^1^, Jin-Young Chung^2^, Ka Yeun Kim^3^, Wooseok Im ^1,4*^, Manho Kim^1,4,5*^

^1^Department of Neurology, Biomedical Research Institute, Seoul National University Hospital, Seoul, South Korea

^2^Department of Veterinary Internal Medicine and Geriatrics, College of Veterinary Medicine, Kangwon National University, Gangwon-do, South Korea

^3^Department of Psychology, Fordham University, New York, NY, United States of America

^4^Neuroscience Research Institute, Seoul National University College of Medicine, Seoul, South Korea

^5^Protein Metabolism Medical Research Center, College of Medicine, Seoul National University Hospital, Seoul, South Korea

**
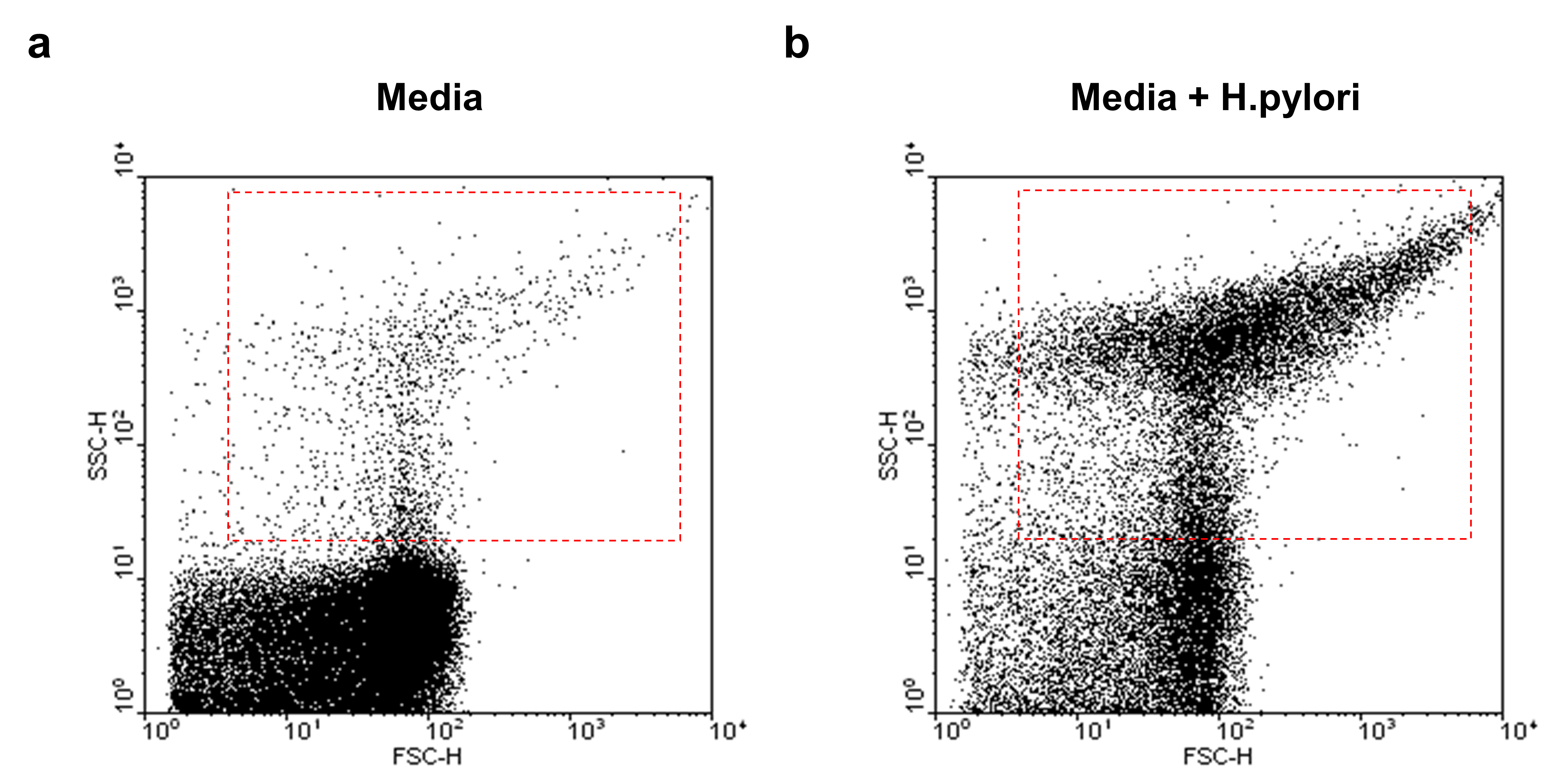
**

**Fig. S1** **Populations of *H.pylori* were analyzed with a flow cytometer.** When the chocolate media was filled with over 90 %, *Helicobacter pylori* was swabbed with sterilized swabs and suspended in RPMI-1640 media 20 ml to form *Helicobacter pylori* suspension. The population in a red gate were assayed as *Helicobacter pylori*. **a** Media, **b** Media + *H. pylori*
